# Primary care utilisation in different patients’ profiles with cardiovascular risk factors

**DOI:** 10.1186/s12913-024-12178-3

**Published:** 2025-01-08

**Authors:** Sara Malo, Lina Maldonado, María José Rabanaque, Irantzu Bengoa-Urrengoechea, Sara Castel-Feced, María Antonia Sánchez-Calavera, Isabel Aguilar-Palacio

**Affiliations:** 1 Department of Preventive Medicine and Public Health, University of Zaragoza, Zaragoza, Spain; 2 Grupo de Investigación en Servicios Sanitarios de Aragón (GRISSA), Fundación Instituto de Investigación Sanitaria de Aragón (IIS Aragón), Zaragoza, Spain; 3 Network for Research On Chronicity, Primary Care, and Health Promotion (RICAPPS), ISCIII, Madrid, Spain; 4 Department of Applied Economics, University of Zaragoza, Zaragoza, Spain; 5 Department of Preventive Medicine and Public Health, Lozano Blesa University Clinical Hospital, Zaragoza, Spain; 6 Department of Statistical Methods, University of Zaragoza, Zaragoza, Spain; 7 Department of Medicine and Psychiatry, University of Zaragoza, Zaragoza, Spain

**Keywords:** Primary health care, Longitudinal study, Risk factors, Spain, Cardiovascular diseases, Big Data, Health care utilization, Predictive medicine

## Abstract

**Background:**

This study aimed to identify profiles of patients with cardiovascular disease (CVD) risk factors, based on their sociodemographic and clinical characteristics, and to analyse how their complexity is related to their frequency of visits in Primary Care.

**Methods:**

Observational longitudinal study conducted in the Spanish CArdiovascular Risk factors for HEalth Services research (CARhES) cohort. Individuals older than 15 with hypertension, type 2 diabetes mellitus (DM) and/or dyslipidaemia in 2017 were selected and followed until 2021. Cluster analyses were performed to identify patients’ profiles according to age, sex and morbidity burden. Characteristics and annual visits in Primary Care in the different profiles were described. Panel data models were applied to study the variability of the frequency of visits to both physicians and nurses in Primary Care in the profiles across different time points.

**Results:**

In this population-based cohort of 446,998 individuals, different profiles were identified among those with hypertension, type 2 DM and/or dyslipidaemia. Profiles comprising the elderly showed the highest morbidity burden. Among the profiles of individuals under 80, those that included women had a higher burden than profiles with men. This higher complexity was associated with higher frequency of Primary Care visits, regardless of the patient’s socioeconomic level and depopulation level of his/her Basic Health Area.

**Conclusions:**

Women and the elderly comprised the profiles with the highest morbidity burden and Primary Care attendance. Tailoring care and resources according to the complexity profile is essential to ensure that patients receive the best possible care based on their needs.

**Supplementary Information:**

The online version contains supplementary material available at 10.1186/s12913-024-12178-3.

## Background

Population ageing leads to a high presence of patients with complex chronic health problems requiring an elevated use of healthcare services. The Primary Care setting is the most suitable level at which the coordination and provision of an appropriate and continuous care for these patients can be ensured, improving health outcomes and reducing subsequent healthcare services utilisation [1].

Cardiovascular disease (CVD) and its risk factors are among the most common health problems, particularly in certain population groups [2]. They have a relevant impact in current societies [3] and require a high need for care which is foreseeably related to the patient complexity. This complexity depends, according to current evidence, on other conditions in addition to traditional CVD risk factors [4].

There is no doubt about the need to tailor the care provided, especially in Primary Care, to the most complex patients’ profiles or groups. In this regard, the Disproportionate Inverse Law is an adaptation of the well-known Inverse Care law to high-income countries. It states that socially disadvantaged people receive more health care, but of poorer quality and in insufficient quantity to meet their additional needs [5]. The secondary use of data from the health system allows to analyse how the care provided to these patients is. This little studied to date issue may provide useful information to improve their management.

This study conducted in a Spanish population-based cohort with CVD risk factors aimed to identify profiles of patients, defined on the basis of their sociodemographic and clinical characteristics, and to analyse its relationship with their frequency of visits in Primary Care during the period 2017–21.

## Methodology

### Study design and population

Observational longitudinal study conducted in the CArdiovascular Risk factors for HEalth Services research (CARhES) cohort. This is a population-based dynamic cohort study of individuals older than 15, resident in Aragón, with hypertension, diabetes mellitus (DM) and/or dyslipidaemia from 2017 onwards. The protocol of the CARhES cohort was approved by the Clinical Research Ethics Committee of Aragon (CEICA PI21/148).

For this study, the population consisted of the CARhES cohort participants with a diagnosis of hypertension, type 2 DM and/or dyslipidaemia, and/or at least one prescription of oral antidiabetic or lipid-lowering therapy, in 2017. They were followed-up until 2021 or until their loss during the study period, due to death or to other causes. Individuals prescribed an antihypertensive drug but without a diagnosis of hypertension were not included, given the widespread use of antihypertensive drugs for numerous pathologies.

Aragón is located in the Northeastern Spain and has almost 1.5 million of inhabitants. The Spanish Healthcare System is mainly funded by taxes and based in universality, free access, equity and fairness of financing. The Primary Care is the core element and constitutes the first contact point with the system, by coordinating the exchange of information among the different health care actors.

Primary health care teams are the basic care structure. They are composed by specialised family physicians and nurses, usually complemented with paediatricians and specialised paediatric nurses, physiotherapists, dentists, psychologists and social workers. Their main functions include diagnostic and therapeutic procedures, preventive measures, health promotion, community care, mental and oral health, and basic rehabilitation.

At the present time, family physicians are the main prescribers and overall supervisors of treatment effectiveness according to established protocols. On the other hand, nurses in Primary Care play a key role in health promotion and disease prevention in chronic patients, and are supervisors of adherence and side-effects [6]. The management of CVD risk factors therefore involves continuous and coordinated action by family physicians, nurses and even other health professionals who perform regular tests, examinations and routine monitoring visits to check adherence, possible adverse effects and the effectiveness of treatment and preventive measures. The number of visits recommended for these patients varies according to the risk factor, their degree of control, the initiation and effects of medication, as well as the occurrence of acute processes or complications [4].

### Data sources and variables of interest

CARhES cohort data were originally obtained from BIGAN [7], a data lake that contains secondary data from the public Healthcare System in Aragón, which represents essentially the entire population. Data from different information systems is linked at the patient level through a pseudonymized individual code: the Users Database (BDU), with sociodemographic information; the Primary Care (OMI-AP) database, with information on visits to Primary Care and the corresponding medical diagnoses; the electronic Dispensation Database, with information on the dispensation of drugs covered by the public Health System; and the Adjusted Morbidity Groups (GMA) database, built based on the diagnostic data collected from the hospital, the Primary and the Emergency Care setting and with information on the presence of specific chronic morbidities for each patient and his/her morbidity burden. The morbidity burden is a summary index based on the clinical conditions present in the patient and the weight assigned to each condition according to the care and resources needed for its management. It was originally developed with data from the Spanish health system and has shown to be associated with the health care needs of patients [8].

Socioeconomic level was determined for each individual through the combination of his/her pharmacy copayment level and type of economic activity, which resulted in seven mutually exclusive categories. The depopulation level of his/her Basic Health Area was determined based on the criteria of the Spanish Ministry for the Ecological Transition and the Demographic Challenge, who defines municipalities depopulated as those with less than 5,000 inhabitants [9]. Clinical characteristics were described based on the information collected in the GMA database for each patient [10]. And pharmacological burden was defined as the number of different pharmacological subgroups [11] that the individual was prescribed and dispensed during the study year.

### Statistical analyses

A 2-step cluster analysis was conducted to identify different profiles among individuals with hypertension, type 2 DM or dyslipidaemia, in Aragón in 2017. Those patients whose morbidity burden information was not available (2.3% of the total) were not included in the profile analyses. This technique automatically determines the optimal number of clusters based on the Bayesian Information Criterion (BIC). In the present study, the variables of interest were the sex, age group and morbidity burden. The quality of fit of the resulting groups was evaluated using the silhouette measure of cohesion and separation.

Characteristics of the study population as well as the number of annual visits to Primary Care physicians and nurses were described, by CVD risk factor and patient’s profile. In order to explore the role of sex in the morbidity burden of each subject, we estimated the frequency of having exact 10 Primary Care visits in both men and women and compared their mean (SD) morbidity burden. Categorical variables were expressed as frequency and proportion, and continuous variables as mean, standard deviation (SD), and also median in the case of the visits.

Univariate and multivariate panel data models were applied to study the variability of the frequency of visits to both physicians and nurses in the different patients’ profiles across different time points (2017–2021). By a random-effects model, the Generalised Least Squares (GLS) method was used as estimator. In the univariate models, the explanatory variable was the patient’s profile and the dependent variable was the number of visits to Primary Care. In the multivariate models, the patient’ socioeconomic level and the depopulation level of his/her Basic Health Area were included as adjustment variables, due to the role of the socioeconomic determinants and environmental exposure as potential CVD risk modifiers [4].

Finally, we calculated the frequency of hospitalisations in both sexes by tertiles of morbidity burden and CVD risk factor.

All analyses were performed using the R Statistical Software (the R Foundation for Statistical Computing, Vienna, Austria), version 4.1.3.

## Results

### Patients’ profiles: identification and characterisation

**Fig. 1:**
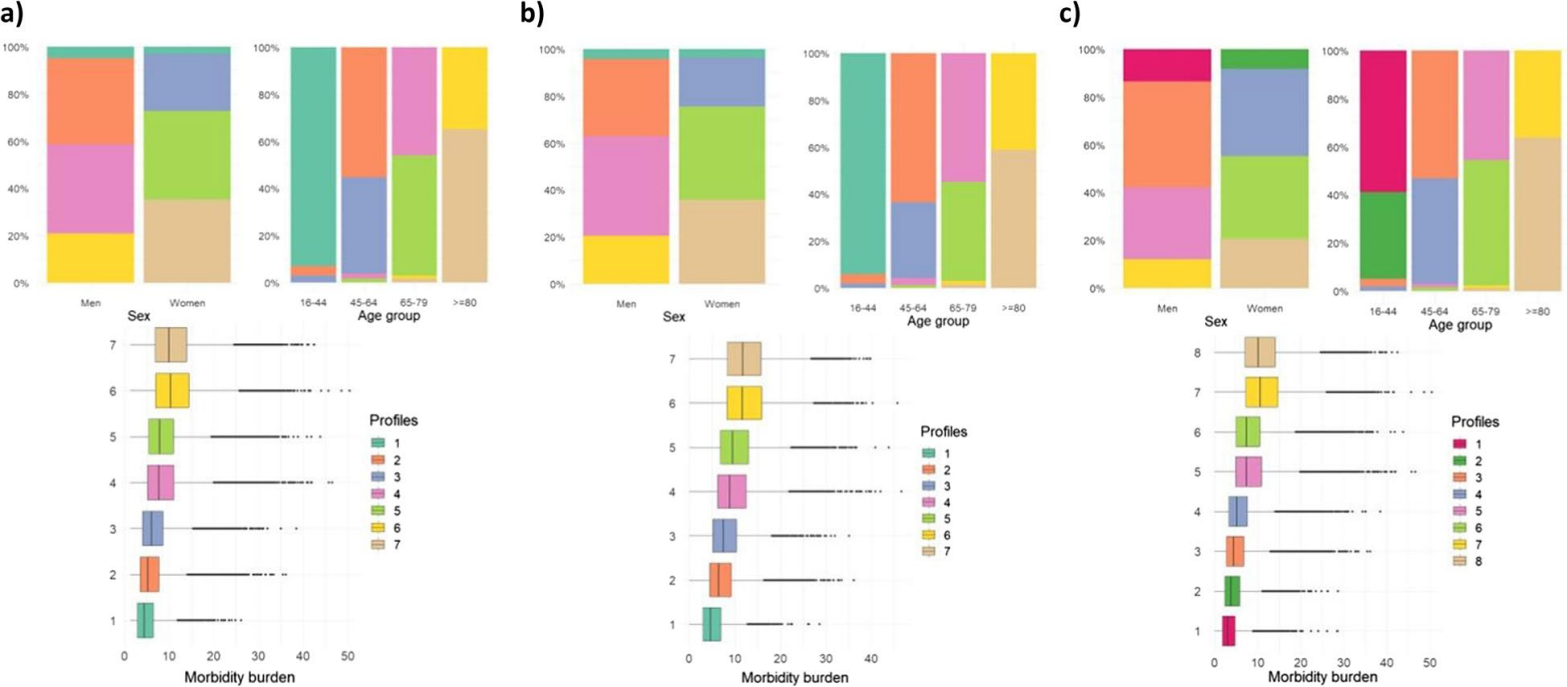
Patients’ profiles identified among those with **a** hypertension, **b** type 2 DM and (**c**) dyslipidaemia. CARhES cohort (Spain), 2017

The original study population consisted of 446,998 individuals: 252,508 had hypertension, 96,709 type 2 DM and 332,644 dyslipidaemia. Individuals with hypertension, type 2 DM and dyslipidaemia were aggregated, respectively, into seven, seven and eight different profiles, defined according to their sex, age group and morbidity burden (Fig. 1). For all three groups, the importance of the three variables was the highest, equal to 1.0, and the silhouette coefficient was 0.8, indicating a high quality of clustering. The patients’ profiles obtained for both hypertension and type 2 DM showed a similar composition: the 1 comprised all the youngest patients (16–44), with a low morbidity burden; 2 and 3 comprised men and women, respectively, in an older age group (45–64); 4 and 5 consisted of men and women, separately, in the following age group (65–79); and finally, 6 and 7 consisted of the oldest (≥ 80) men and women, respectively. In all the cases, when comparing profiles composed of patients in the same age group, those comprising women had higher morbidity burden.

Regarding patients with dyslipidaemia, the composition of the profiles was similar, with the exception that younger patients (16–44) were divided into two profiles, according to sex. Again, the profiles comprising women showed the highest morbidity burden.

Sociodemographic and clinical characteristics of patients in the identified profiles are shown in Table 1 (hypertension), Table 2 (type 2 DM) and Table 3 (dyslipidaemia). Most of the study patients, particularly the youngest profiles, lived in a Health Care Area with no depopulated municipalities, i.e., an urban area. The most frequent comorbidities were obesity, especially in the profiles comprising patients in the 45–64 and 65–79 age groups; depression, with a frequency higher in women in the oldest age groups; and chronic kidney disease, more common in all profiles from 65 years old onwards. Morbidity and pharmacological burden increased with age, being usually higher in profiles comprising women.

**Table 1:** Sociodemographic and clinical characteristics of individuals with hypertension (*N* = 252,444) included in each profile. CARhES cohort, 2017

	**Profile 1**	**Profile 2**	**Profile 3**	**Profile 4**	**Profile 5**	**Profile 6**	**Profile 7**	**Total**
**Sociodemographic characteristics**								
Sex, n (%)								
Men	5,920 (4.9%)	43,626 (36.4%)	0%	45,259 (37.8%)	0%	24,975 (20.9%)	0%	119,780 (100%)
Women	3,858 (2.9%)	0%	32,323 (24.4%)	0%	49,842 (37.6%)	0%	46,641 (35.2%)	132,664 (100%)
Age group, n (%)								
16–44	9,778 (92.9%)	437 (4.2%)	309 (2.9%)	0%	0%	0%	0%	10,524 (100%)
45–64	0%	43,189 (55.2%)	32,014 (41.0%)	1591 (2.0%)	1382 (1.8%)	1 (≈0%)	0%	78,177 (100%)
65–79	0%	0%	0%	43,668 (41.0%)	48,460 (51.1%)	1,074 (1.1%)	1,721 (1.8%)	94,923 (100%)
≥ 80	0%	0%	0%	0%	0%	23,900 (34.7%)	44,920 (65.3%)	68,820 (100%)
Socioeconomic level, n (%)								
Active < 18K	4,886 (16.1%)	13,106 (43.2%)	11,267 (37.2%)	324 (1.1%)	698 (2.3%)	13 (≈0%)	31 (0.1%)	30,325 (100%)
Active ≥ 18K	2,357 (10.4%)	14,353 (63.5%)	5,352 (23.7%)	271 (1.2%)	243 (1.1%)	5 (≈0%)	8 (≈0%)	22,589 (100%)
Unemployed	649 (11.9%)	2,542 (44.4%)	2,350 (41.1%)	43 (0.8%)	104 (1.8%)	1 (≈0%)	1 (≈0%)	5,720 (100%)
Mutualist	39 (2.2%)	331 (18.8%)	252 (14.3%)	403 (22.8%)	341 (19.3%)	143 (8.1%)	256 (14.5%)	1,765 (100%)
Pensioner < 18K	859 (0.6%)	7,792 (5.6%)	7,447 (5.4%)	25,519 (18.3%)	36,324 (26.1%)	19,777 (14.2%)	41,468 (29.8%)	139,186 (100%)
Pensioner ≥ 18K	55 (0.1%)	4,075 (8.8%)	3,113 (6.7%)	18,379 (39.6%)	11,377 (24.5%)	4,945 (10.6%)	4,522 (9.7%)	46,466 (100%)
Other	903 (14.1%)	1,427 (22.3%)	2,542 (39.8%)	320 (5%)	755 (11.8%)	91 (1.42%)	355 (5.6%)	6,393 (100%)
Depopulation level, n (%)								
0% depopulated municipalities	5,720 (4.0%)	24,598 (17.4%)	18,981 (13.4%)	25,872 (18.3%)	29,451 (20.8%)	12,081 (8.5%)	24,878 (17.6%)	141,581 (100%)
Some depopulated municipalities	2,853 (4.4%)	11,868 (18.4%)	8,419 (13.1%)	10,905 (16.9%)	11,566 (18.0%)	6,704 (10.4%)	12,102 (18.8%)	64,417 (100%)
100% depopulated municipalities	1,205 (2.6%)	7,160 (15.4%)	4,923 (10.6%)	8,482 (18.3%)	8,825 (19.0%)	6,190 (13.3%)	9,661 (20.8%)	46,446 (100%)
**Clinical characteristics**								
Comorbidities presence, n (%)								
Heart failure	33 (0.3%)	463 (3.9%)	223 (1.9%)	1,550 (13.1%)	1,546 (13.1%)	2,653 (22.5%)	5,343 (45.2%)	11,811 (100%)
COPD	80 (0.5%)	2,024 (11.6%)	920 (5.3%)	5,668 (32.5%)	2,215 (12.7%)	4,124 (23.6%)	2,411 (13.8%)	17,442 (100%)
Depression	884 (2.1%)	3,710 (9%)	6,544 (15.9%)	3,846 (9.3%)	11,913 (28.9%)	2,647 (6.4%)	11,703 (28.4%)	41,247 (100%)
Ischemic cardiopathy	105 (0.5%)	2,700 (13.6%)	690 (3.5%)	5,579 (28.1%)	2,594 (13.1%)	4,024 (20.3%)	4,177 (21%)	19,869 (100%)
Stroke	67 (0.5%)	1,054 (8.5%)	512 (4.1%)	2,526 (20.3%)	1,771 (14.2%)	2,619 (21.1%)	3,880 (31.2%)	12,429 (100%)
Chronic renal disease	873 (2.2%)	4,290 (10.9%)	2,545 (6.5%)	7,416 (18.9%)	6,286 (16.0%)	6,550 (16.7%)	11,369 (28.9%)	39,329 (100%)
Dementia	5 (0.1%)	53 (0.6%)	44 (0.5%)	643 (6.8%)	1,023 (10.9%)	1,773 (18.9%)	5,850 (62.3%)	9,391 (100%)
Obesity	2,441 (4.9%)	8,757 (17.6%)	7,945 (15.9%)	8,189 (16.4%)	11,719 (23.5%)	3,107 (6.2%)	7,668 (15.4%)	49,826 (100%)
Morbidity burden, mean (SD)	5.0 (3.0)	6.0 (3.6)	6.7 (3.7)	8.6 (4.9)	8.7 (4.6)	11.2 (5.8)	10.8 (5.4)	8.5 (5.0)
Pharmacological burden, mean (SD)	4.2 (3.5)	5.2 (3.7)	6.6 (4.3)	4.6 (4.3)	8.9 (4.7)	9.1 (4.5)	9.7 (4.6)	7.7 (4.7)

*COPD* Chronic Obstructive Pulmonary Disease, *SD* Standard Deviation

**Table 2:** Sociodemographic and clinical characteristics of individuals with type 2 DM (*N* = 71,951) included in each profile. CARhES cohort, 2017

	**Profile 1**	**Profile 2**	**Profile 3**	**Profile 4**	**Profile 5**	**Profile 6**	**Profile 7**	**Total**
**Sociodemographic characteristics**								
Sex, n (%)								
Men	1,657 (4.2%)	13,143 (33.1%)	0%	16,817 (42.3%)	0%	8,147 (20.5%)	0%	39,764 (100%)
Women	1,192 (3.7%)	0%	6,688 (20.8%)	0%	12,786 (39.7%)	0%	11,521 (35.8%)	32,187 (100%)
Age group, n (%)								
16–44 y.o	2,849 (94.1%)	118 (3.9%)	62 (2.0%)	0%	0%	0%	0%	3,029 (100%)
45–64 y.o	0%	13,025 (63.5%)	6,626 (32.3%)	573 (2.8%)	287 (1.4%)	0%	0%	20,511 (100%)
65–79 y.o	0%	0%	0%	16,244 (54.9%)	12,499 (42.2%)	412 (1.4%)	458 (1.6%)	29,613 (100%)
≥ 80 y.o	0%	0%	0%	0%	0%	7,735 (41.0%)	11,063 (59.0%)	18,798 (100%)
Socioeconomic level, n (%)								
Active < 18K	1,422 (18.7%)	3,840 (50.4%)	2,073 (27.2%)	134 (1.8%)	136 (1.8%)	5 (0.1%)	6 (0.1%)	7,616 (100%)
Active ≥ 18K	552 (11.5%)	3,338 (69.6%)	776 (16.2%)	83 (1.7%)	46 (1.0%)	2 (0.04%)	1 (0.02%)	4,798 (100%)
Unemployed	187 (12.6%)	814 (54.6%)	958 (34.3%)	15 (1.0%)	23 (1.5%)	0%	0%	1,490 (100%)
Mutualist	11 (3.0%)	62 (17.1%)	29 (8.0%)	101 (27.9%)	62 (17.1%)	44 (12.2%)	53 (14.6%)	362 (100%)
Pensioner < 18K	373 (0.9%)	3,338 (7.8%)	2,112 (5.0%)	10,120 (23.8%)	9,806 (23.0%)	6,382 (15.0%)	10,442 (24.5%)	42,573 (100%)
Pensioner ≥ 18K	29 (0.2%)	1,270 (9.5)	674 (5.0%)	6,256 (46.7%)	2,522 (18.8%)	1,693 (12.7%)	940 (7.0%)	13,384 (100%)
Other	275 (15.9%)	481 (27.8%)	573 (33.2%)	108 (6.2%)	191 (11.1%)	21 (1.2%)	79 (4.6%)	1,728 (100%)
Depopulation level, n (%)								
0% depopulated municipalities	1,726 (4.3%)	7,204 (19.9%)	3,995 (9.9%)	9,639 (23.9%)	7,426 (18.4%)	4,176 (10.3%)	6,189 (15.3%)	40,355 (100%)
Some depopulated municipalities	768 (4.2%)	3,665 (19.9%)	1,697 (9.2%)	4,038 (21.9%)	3133 (17%)	2,177 (11.8%)	2,979 (16.1%)	18,457 (100%)
100% depopulated municipalities	355 (2.7%)	2,274 (17.3%)	996 (7.6%)	3,140 (23.9%)	2,227 (16.9%)	1,794 (13.7%)	2,353 (17.9%)	13,139 (100%)
**Clinical characteristics**								
Comorbidities presence, n (%)								
Heart failure	12 (0.3%)	247 (5.4%)	98 (2.2%)	860 (19.0%)	645 (14.2%)	1,020 (22.5%)	1,653 (36.4%)	4,535 (100%)
COPD	33 (0.6%)	787 (13.5%)	246 (4.2%)	2,194 (37.5%)	605 (10.3%)	1,384 (23.7%)	598 (10.2%)	5,847 (100%)
Depression	280 (2.3%)	1,239 (10.4%)	1,586 (13.3%)	1,510 (12.7%)	3,353 (28.1%)	939 (7.9%)	3,024 (25.3%)	11,931 (100%)
Ischemic cardiopathy	31 (0.4%)	1,314 (15.4%)	230 (2.7%)	2,814 (33.6%)	1,059 (12.6%)	1,620 (19.3%)	1,314 (15.7%)	8,382 (100%)
Stroke	15 (0.3%)	371 (8.6%)	136 (3.1%)	1,085 (25.1%)	602 (13.9%)	974 (22.5%)	1,138 (26.3%)	4,321 (100%)
Chronic renal disease	77 (0.7%)	1,106 (9.9%)	448 (4.0%)	2,713 (24.2%)	1,803 (16.1%)	2,075 (18.5%)	3,004 (26.8%)	11,226 (100%)
Dementia	1 (0.03%)	23 (0.8%)	17 (0.6%)	303 (10.5%)	372 (12.9%)	641 (22.1%)	1,537 (53.1%)	2,894 (100%)
Obesity	558 (3.3%)	3,107 (18.4%)	2,255 (13.3%)	3,512 (20.8%)	3,929 (23.2%)	1,178 (7.0%)	2,363 (14.0%)	16,902 (100%)
Morbidity burden, mean (SD)	5.4 (3.3)	7.3 (4.1)	8.2 (4.2)	9.9 (5.2)	10.4 (5.0)	12.6 (5.9)	12.5 (5.6)	9.9 (5.4)
Pharmacological burden, mean (SD)	4.8 (3.6)	6.4 (4.1)	8.4 (4.9)	8.7 (4.5)	10.5 (4.9)	10.3 (4.7)	11.1 (4.7)	9.0 (4.9)

*COPD* Chronic Obstructive Pulmonary Disease, *SD* Standard Deviation

**Table 3:** Sociodemographic and clinical characteristics of individuals with dyslipidaemia (*N* = 328,430) included in each profile. CARhES cohort, 2017

	**Profile 1**	**Profile 2**	**Profile 3**	**Profile 4**	**Profile 5**	**Profile 6**	**Profile 7**	**Profile 8**	**Total**
**Sociodemographic characteristics**									
Sex, n (%)									
Men	22,343 (13.6%)	0%	72,600 (44.2%)	0%	49,524 (30.2%)	0%	19,660 (12.0%)	0%	164,127 (100%)
Women	0%	13,678 (8.3%)	0%	59,813 (36.4%)	0%	56,645 (34.5%)	0%	34,167 (20.8%)	164,303 (100%)
Age group, n (%)									
16–44 y.o	22,343 (58.8%)	13,678(36.0%)	1,267 (3.3%)	701 (1.8%)	0%	0%	0%	0%	37,989 (100%)
45–64 y.o	0%	0%	71,333 (53.1%)	59,112 (44%)	1,969 (1.5%)	2,033 (1.5%)	2 (≈0%)	0%	134,449 (100%)
65–79 y.o	0%	0%	0%	0%	47,555 (45.5%)	54,612 (52.2%)	964 (0.9%)	1,471 (1.4%)	104,602 (100%)
≥ 80 y.o	0%	0%	0%	0%	0%	0%	18,694 (36.4%)	32,696 (63.6%)	51,390 (100%)
Socioeconomic level, n (%)									
Active < 18K	10,762 (16.6%)	7,631 (11.8%)	23,256 (36.0%)	21,769 (33.7%)	381 (0.6%)	860 (1.3%)	9 (≈0%)	19 (≈0%)	64,687 (100%)
Active ≥ 18K	7,334 (15.6%)	2,355 (5%)	25,081 (53.3%)	11,613 (24.7%)	314 (0.7%)	341 (0.7%)	8 (≈0%)	8 (≈0%)	47,054(100%)
Unemployed	1,143 (10.8%)	1,039 (9.8%)	3,979 (37.4%)	4,280 (40.3%)	46 (0.4%)	140 (1.3%)	0%	1 (≈0%)	10,628 (100%)
Mutualist	92 (4.0%)	97 (4.2%)	526 (22.7%)	582 (25.1%)	391 (16.9%)	388 (16.7%)	79 (3.4%)	165 (7.1%)	2,320 (100%)
Pensioner < 18K	1,402 (1.0%)	1,108 (0.8%)	11,787 (8.5%)	11,794 (8.5%)	27,373 (19.8%)	39,472 (28.6%)	15,360 (11.1%)	29,956 (21.7%)	138,252 (100%)
Pensioner ≥ 18K	165 (0.3%)	94 (0.2%)	5,751 (10.5%)	5,409 (9.9%)	20,717 (37.9%)	14,675 (26.8%)	4,130 (7.6%)	3,769 (6.9%)	54,710 (100%)
Other	1,445 (13.4%)	1,354 (12.6%)	2,220 (20.6%)	4,366 (40.5%)	302 (2.8%)	769 (7.1%)	74 (0.7%)	249 (2.3%)	10,779 (100%)
Depopulation level, n (%)									
0% depopulated municipalities	12,812 (6.7%)	8,419 (4.4%)	40,328 (21.2%)	36,013 (19%)	28,836 (15.2%)	35,056 (18.5%)	9,786 (5.2%)	18,657 (9.8%)	189,907 (100%)
Some depopulated municipalities	6,346 (7.6%)	3,558 (4.3%)	19,830 (23.8%)	15,083 (18.1%)	11,788 (14.2%)	12,539 (15.1%)	5,259 (6.3%)	8,859 (10.6%)	83,262 (100%)
100% depopulated municipalities	3,185 (5.8%)	1,701 (3.1%)	12,442 (22.5%)	8,717 (15.8%)	8,900 (16.1%)	9,050 (16.4%)	4,615 (8.4%)	6,651 (12%)	55,261 (100%)
**Clinical characteristics**									
Comorbidities presence, n (%)									
Heart failure	35 (0.3%)	20 (0.2%)	570 (5.7%)	328 (3.3%)	1,650 (16.5%)	1,525 (15.2%)	2,136 (21.4%)	3,740 (37.4%)	10,004 (100%)
COPD	130 (0.7%)	46 (0.2%)	2,956 (16.1%)	1,626 (8.8%)	6,120 (33.2%)	2,459 (13.4%)	3,299 (17.9%)	1,775 (9.6%)	18,411 (100%)
Depression	1,437 (2.8%)	1,624 (3.2%)	6,270 (12.2%)	12,404 (24.1%)	4,512 (8.8%)	13,948 (27.1%)	2,201 (4.3%)	9,071 (17.6%)	51,467 (100%)
Ischemic cardiopathy	281 (1.0%)	66 (0.6%)	5,434 (20.2%)	1,215 (4.5%)	8,138 (30.2%)	3,179 (11.8%)	4,808 (17.8%)	3,822 (14.2%)	26,943 (100%)
Stroke	95 (0.7%)	53 (0.4%)	1,494 (11.6%)	775 (6.0%)	2,915 (22.7%)	1,944 (15.1%)	2,466 (19.2%)	3,118 (24.2%)	12,860 (100%)
Chronic renal disease	284 (1.0%)	130 (0.4%)	3,376 (11.4%)	1,831 (6.2%)	6,336 (21.3%)	5,241 (17.6%)	4,735 (15.9%)	7,768 (26.2%)	29,701 (100%)
Dementia	11 (0.1%)	3 (≈0%)	93 (1.2%)	98 (1.3%)	725 (9.2%)	1,179 (15.0%)	1,458 (18.6%)	4,272 (54.5%)	7,839 (100%)
Obesity	2,322 (4.8%)	1,687 (3.5%)	9,622 (20.1%)	8,756 (18.3%)	7,513 (15.7%)	10,336 (21.6%)	2,331 (4.9%)	5,370 (11.2%)	47,937 (100%)
Morbidity burden, mean (SD)	3.6 (2.4)	4.4 (2.9)	5.2 (3.5)	5.8 (3.5)	8.3 (4.9)	8.2 (4.5)	11.4 (5.8)	10.9 (5.4)	7.1 (4.8)
Pharmacological burden, mean (SD)	2.9 (2.5)	4.1 (3.4)	4.5 (3.6)	5.8 (4.1)	7.5 (4.3)	8.5 (4.7)	9.6 (4.5)	10.0 (4.6)	6.6 (4.6)

*COPD* Chronic Obstructive Pulmonary Disease, *SD* Standard Deviation

### Frequency of visits to Primary Care physicians and nurses

In 2017, most of the patients with hypertension, type 2 DM and/or dyslipidaemia visited a physician and/or a nurse at least once. There were few differences between men and women. The coincidence of the date of the visit to the physician and the nurse was higher in patients with type 2 DM patients than for those with hypertension or dyslipidaemia. Thus, 63.3% of men with type 2 DM and 69.9% of women with type 2 DM visited the physician and the nurse at least once on the same date (Table 4).

**Table 4:** Frequency of patients with visits to the Primary Care physician, the nurse or both. Analyses conducted separately for patients with the different CVD risk factors and by sex. CARhES cohort, 2017

		**Patients with visits to physician and/or nurse**	**Patients with visits only to physician**	**Patients with visits only to nurse**	**Patients with visits to both on the same date**
**Hypertension**	*Total*	247,060 (97.8)	244,263 (96.7)	187,306 (74.2)	149,264 (59.1)
	*Men*	116,658 (97.4)	115,193 (96.1)	85,492 (71.4)	66,440 (55.5)
	*Women*	130,402 (98.3)	129,070 (97.3)	101,814 (76.7)	82,824 (62.42)
**Type 2 DM**	*Total*	94,959 (98.2)	93,758 (97.0)	79,745 (82.5)	64,096 (66.3)
	*Men*	51,889 (98.2)	51,176 (96.8)	42,930 (81.2)	33,438 (63.3)
	*Women*	43,070 (98.2)	42,582 (97.1)	36,815 (84.0)	30,658 (69.9)
**Dyslipidaemia**	*Total*	321,042 (96.5)	317,527 (95.5)	214,075 (64.4)	178,114 (53.5)
	*Men*	158,754 (95.3)	156,621 (94.1)	101,724 (61.1)	83,232 (50.0)
	*Women*	162,288 (97.7)	160,906 (96.9)	112,351 (67.6)	94,882 (57.1)

Frequency expressed as N (%)

*DM* Diabetes Mellitus

A detailed description of the annual frequency of visits (2017–2021) to both physicians and nurses, for the three CVD risk factors and the corresponding profiles, is presented in the Supplementary Table 1 (Table S1). The frequency of visits increased with increasing patients’ age and morbidity burden. Moreover, the highest number of visits to the physician was observed, among the profiles comprising the individuals younger than 80, in those composed of women. Above 80 years of age, the frequency was similar for men and women. Sex differences were lower in the case of visits to the nurse. During 2020 and 2021, the younger patients’ profiles showed an increase in the mean number of visits to Primary Care while among older patients, the mean frequency slightly decreased in 2020 and then recovered to the previous values or remained the same.

To further explore the differences found between men and women, Table 5 shows the frequency of individuals with 10 visits to the family physician, as well as their mean (SD) morbidity burden. A slightly higher proportion of women than men visited the Primary Care physician 10 times during 2017, for the three CVD risk factors. Among these people, the mean morbidity burden was very similar between both sexes.

**Table 5:** Frequency of individuals, by sex and by CVD risk factor, with 10 visits to the family physician, and their mean (SD) morbidity burden. CARhES cohort, 2017

		**Total**	**Hypertension**	**Type 2 DM**	**Dyslipidaemia**
**N (%) patients with 10 visits to physician**	*Total*	20,895 (4.7)	12,407 (4.9)	3,314 (3.4)	15,728 (4.7)
	*Men*	9,213 (4.2)	5,395 (4.5)	1,670 (3.2)	7,105 (4.3)
	*Women*	11,682 (5.2)	7,012 (5.3)	1,644 (3.7)	8,623 (5.2)
**Morbidity burden, mean (SD)**	*Total*	7.9 (4.1)	9.1 (4.1)	10.9 (4.3)	7.8 (4.1)
	*Men*	7.8 (4.3)	9.0 (4.3)	10.8 (4.4)	7.8 (4.2)
	*Women*	7.9 (4.0)	9.1 (4.0)	11.1 (4.1)	7.8 (3.9)

Frequency expressed as N (%)

*DM* Diabetes Mellitus, *SD* Standard Deviation

In supplementary analyses (Table S2), it was observed that among subjects with low morbidity burden, women were more likely to be hospitalised, but as morbidity burden increased, men were more likely to be hospitalised.

### Association between patients’ profiles and frequency of visits to Primary Care

The panel data analyses confirmed the results obtained in the descriptive analysis. For the three CVD risk factors, the frequency of visits to both physicians (Table 6) and nurses (Table 7) increased as the complexity, i.e., the age and morbidity burden, of the patients increased. This association was also observed after adjusting by patient’ socioeconomic level and depopulation level of the Basic Health Area of residence. With regard to the visits to physicians, the elderly showed, on average, about 2.8 (hypertension), 2.5 (type 2 DM) and 4.7 (dyslipidaemia) more visits than patients in profile 1, who are the youngest. Moreover, profiles composed of women (3, 5 and 7 for hypertension and type 2 DM, and 2, 4, 6 and 8 for dyslipidaemia) showed a higher frequency of visits than profiles composed of men in the same age groups. This was found for all the profiles except for those ≥ 80 years (profiles 6 and 7 for hypertension and type 2 DM, and 7 and 8 for dyslipidaemia), where the frequency of visits in men and women was similar. With regard to the visits to nurses, differences between the more and the less complex profiles were even higher. However, differences by sex were lower.

**Table 6:** Influence of patient’s profile on the frequency of visits to Primary Care physicians. Analyses conducted separatedly for patients with the different CVD risk factors

CVD risk factor	Profiles	Univariate estim. (95% CI)	*p*-value	Multivariate estim. (95% CI)	*p*-value
Hypertension	1	-	-	-	-
	2	0.3 (0.3 – 0.4)	< 0.001	0.1 (0.0 – 0.2)	0.037
	3	2.1 (2.1 – 2.2)	< 0.001	1.9 (1.8 – 1.9)	< 0.001
	4	1.6 (1.5 – 1.6)	< 0.001	0.7 (0.8 – 0.8)	< 0.001
	5	3.0 (2.9 – 3.1)	< 0.001	1.9 (1.9 – 2.0)	< 0.001
	6	4.2 (4.1 – 4.3)	< 0.001	2.8 (2.7 – 2.9)	< 0.001
	7	4.2 (4.1 – 4.2)	< 0.001	2.8 (2.7 – 2.9)	< 0.001
Type 2 DM	1	-	-	-	-
	2	0.3 (0.2 – 0.5)	< 0.001	−0.1 (−0.3 – 0.0)	0.157
	3	2.5 (2.3 – 2.7)	< 0.001	2.1 (2.0 – 2.3)	< 0.001
	4	1.4 (1.2 – 1.6)	< 0.001	0.4 (0.3 – 0.6)	< 0.001
	5	3.2 (3.0 – 3.3)	< 0.001	2.0 (1.9 – 2.2)	< 0.001
	6	3.9 (3.7 – 4.0)	< 0.001	2.5 (2.3 – 2.7)	< 0.001
	7	4.0 (3.9 – 4.2)	< 0.001	2.6 (2.4 – 2.8)	< 0.001
Dyslipidaemia	1	-	-	-	-
	2	2.3 (2.2 – 2.4)	< 0.001	2.3 (2.2 – 2.4)	< 0.001
	3	1.7 (1.6 – 1.7)	< 0.001	1.5 (1.4 – 1.5)	< 0.001
	4	3.5 (3.4 – 3.5)	< 0.001	3.2 (3.2 – 3.3)	< 0.001
	5	3.3 (3.2 – 3.3)	< 0.001	2.3 (2.3 – 2.4)	< 0.001
	6	4.4 (4.4 – 4.5)	< 0.001	3.4 (3.4 – 3.5)	< 0.001
	7	6.1 (6.0 – 6.2)	< 0.001	4.7 (4.6 – 4.8)	< 0.001
	8	6.0 (6.0 – 6.1)	< 0.001	4.6 (4.6 – 4.7)	< 0.001

Analysis of panel data adjusted by socioeconomic level of the patient and depopulation level of his/her Basic Health Area

*CI* Confidence Interval, *DM* Diabetes Mellitus

**Table 7:** Influence of patient’s profile on the frequency of visits to Primary Care nurses. Analyses conducted separatedly for patients with the different CVD risk factors

CVD risk factor	Profiles	Univariate estim. (95% CI)	*p*-value	Multivariate estim. (95% CI)	*p*-value
Hypertension	1	-	-	-	-
	2	1.1 (1.0 – 1.2)	< 0.001	0.6 (0.5 – 0.7)	< 0.001
	3	1.5 (1.4 – 1.6)	< 0.001	0.8 (0.7 – 0.9)	< 0.001
	4	4.4 (4.3 – 4.5)	< 0.001	2.4 (2.3 – 2.6)	< 0.001
	5	4.4 (4.3 – 4.5)	< 0.001	2.2 (2.1 – 2.3)	< 0.001
	6	7.6 (7.5 – 7.7)	< 0.001	5.1 (5.0 – 5.2)	< 0.001
	7	6.6 (6.5 – 6.7)	< 0.001	4.1 (4.0 – 4.2)	< 0.001
Type 2 DM	1	-	-	-	-
	2	−0.2 (−0.4 – 0.1)	0.157	−1.0 (−1.3 – −0.8)	< 0.001
	3	0.5 (0.3 – 0.8)	< 0.001	−0.4 (−0.7 – −0.2)	< 0.001
	4	2.9 (2.7 – 3.1)	< 0.001	0.5 (0.2 – 0.7)	< 0.001
	5	3.4 (3.2 – 3.7)	< 0.001	0.8 (0.5 – 1.0)	< 0.001
	6	5.8 (5.5 – 6.0)	< 0.001	2.9 (2.6 – 3.1)	< 0.001
	7	5.4 (5.1 – 5.6)	< 0.001	2.3 (2.1 – 2.6)	< 0.001
Dyslipidaemia	1	-	-	-	-
	2	0.4 (0.4 – 0.5)	< 0.001	0.4 (0.3 – 0.5)	< 0.001
	3	1.4 (1.3 – 1.4)	< 0.001	0.9 (0.9 – 1.0)	< 0.001
	4	1.5 (1.4 – 1.6)	< 0.001	1.0 (0.9 – 1.0)	< 0.001
	5	4.8 (4.8 – 4.9)	< 0.001	3.0 (3.0 – 3.1)	< 0.001
	6	4.4 (4.3 – 4.4)	< 0.001	2.4 (2.4 – 2.5)	< 0.001
	7	8.3 (8.3 – 8.4)	< 0.001	6.1 (6.0 – 6.1)	< 0.001
	8	7.2 (7.1 – 7.2)	< 0.001	4.8 (4.8 – 4.9)	< 0.001

Analysis of panel data adjusted by socioeconomic level of the patient and depopulation level of his/her Basic Health Area

*DM* Diabetes Mellitus

## Discussion

### Summary

In this population-based cohort study of individuals with traditional CVD risk factors, patients’ profiles comprising the elderly and women showed a higher morbidity burden, possibly indicating a greater need for care. Moreover, the results of the adjusted analysis showed a higher frequency of visits to Primary Care in these profiles consisting of elderly and women, up to the age of 80 years when the frequency is equalised with men. This was found for the entire period analysed and regardless of the patient’s socioeconomic level and the depopulation level of his/her Basic Health Area.

### Comparison with existing literature

Current European guidelines on CVD prevention [4] present age as the major driver of CVD risk, and sex as a risk modifying factor that leads to specific clinical interventions, and emphasise the role of other potential risk modifiers (e.g., frailty or socioeconomic determinants) and clinical conditions (e.g., chronic renal disease or heart failure). In our analyses, both age and sex determined the aggregation of patients into the profiles defined, together with the morbidity burden, leading to a higher presence of some clinical conditions related to CVD in the more complex patients’ profiles, for the three CVD risk factors. In this regard, the inequities found in the frequency of visits in Primary Care, with the older profiles with a higher frequency, is aligned with the expected, as more complex patients tend to require more intensive care. In relation to sex differences, the greater frequency observed in the profiles composed of women, compared with those with men at the same age, coincides with previous research [12–14]. However, these differences disappeared after the age of 80 years. One possible explanation could be that women below this age usually have a poorer perceived health status and a higher prevalence of affective disorders, arthritis or other processes affecting their quality of life, compared to men [14]. Women also tend to be more adherent to recommendations regarding routine medical controls, having shown a greater use of both telephone and in-person medical consultations during the pandemic. In contrast, men with high complexity may have visited the nurse more frequently than women with the same risk, due to a lower capacity for self-care [13].

One might think that women, because they go to the family physician more often, are more likely to get a clinical diagnosis and therefore have a higher morbidity burden. However, it is reasonable to assume that chronic disease diagnoses typically occur during a patient's initial medical visits, making the total number of visits less significant. Our estimates support this, revealing that although women appear to visit Primary Care more frequently, their morbidity burden is similar to that of male frequent attenders. Additionally, the lower use of health services other than Primary Care in women compared with men is well known [15]. This is partly supported by our results, where a slightly lower frequency of hospitalisation was found in women, but only when they had a high disease burden. Further research is needed to understand the reasons for and consequences of gender inequities in health services use and outcomes. Also, analysing the effect of the frequency of visits observed in each profile on the CVD risk factors control, the occurrence of a cardiovascular event, or the use of other health services, such as emergencies or hospitalisations are priority lines.

Clinical practice guidelines on multimorbidity management and associated problems, such as polypharmacy and adherence, are considered scarce [16, 17]. Most of them continue being focused on the prevention of the main risk factors for one specific disease. In the research field, the number of studies on multimorbidity has been growing in recent years but, to our knowledge, there are still very few research that classify the study population by complexity profiles [14, 18–20]. It seems key when it concerns the analysis of very different realities in patients with several diagnoses. Their classification according to their risk or complexity can help health care professionals in routine clinical practice to take decisions on the resources, the intensity or the frequency of the care to be provided to meet their individual needs. Evidence in this field can also provide policy-makers with useful information to develop public health policies that allocate more and better adapted resources to populations with the greatest needs, in this case women and the elderly, from an equitable perspective.

### Strengths and limitations

The main strengths are related to the study population and sources of information. The cohort analysed comprises all individuals with at least one CVD risk factor in a Spanish Autonomous Community considered as a broadly representative population of the country. Moreover, the integration and analysis of large population-based databases linking individual, clinical and drug information provide knowledge on the access to Primary Care professionals by different patients’ groups within the same population. Few studies to date have shown a similar approach.

In terms of study limitations, the identification of individuals in the study cohort as having type 2 DM or dyslipidaemia is based on their clinical diagnosis or medication. Although this may lead to some misclassification, it is considered a highly sensitive inclusion criterion. Also, one patient could have been included in two or three of the groups compared, e.g., presenting hypertension and dyslipidaemia simultaneously. However, the consideration of morbidity burden when defining the profiles may mitigate possible misinterpretation of the results, as this burden index is strongly influenced by the presence of a CVD risk factor diagnosis. Regarding private healthcare, the CARhES cohort does not include visits to primary care professionals. This however represents a minor use in a public health system such as the Spanish one, with universal coverage. The limitations related to the data sources used, such as the quality of the data, could influence the findings. Anyway, these sources have been used in numerous previous studies and are routinely subjected to quality control and debugging. Finally, having information on the type of consultation (e.g., in-person/telephone) or the reason for each visit (e.g., related to the CVD risk factor or to a COVID-19 diagnosis) would have been useful to understand some findings, such as the higher frequency of visits to physicians compared to nurses.

## Conclusions

Our findings allow to recognise different profiles among patients with CVD risk factors according to their complexity and needs, as well as differences between the profiles in the attendance to the Primary Care physician and nurse. We also demonstrated that complex and multimorbid patients are usually exposed to a high pharmacy burden.

Understanding how patients with complex conditions and multimorbidity use Primary Care can inform necessary changes in health systems, helping to reduce deficiencies and improve future responses.

## Supplementary Information

Supplementary Material 1.

## Data Availability

The data analyzed in this study is subject to the following licenses/restrictions: All data used in this study pertain to the CARhES cohort. While these data are not publicly available due to their sensitive nature, interested researchers can nonetheless contact the Principal Investigators (Sara Malo and Isabel Aguilar-Palacio) to request access. Requests to access these datasets should be directed to smalo@unizar.es and iaguilar@unizar.es.

## Abbreviations

CVD: Cardiovascular disease
DM: Diabetes mellitus
CARhES cohort: CArdiovascular Risk factors for HEalth Services research cohort
BIC: Bayesian Information Criterion
SD: Standard deviation
GLS method: Generalised Least Squares method
