# Supplementary material for: Primary care utilisation in different patients’ profiles with cardiovascular risk factors
Source: BMC Health Serv Res. 2025 Jan 8;25:42. doi: 10.1186/s12913-024-12178-3 (PMC11707849; doi:10.1186/s12913-024-12178-3)
Supplement: Supplementary file 1 — Supplementary Material 1. [file 12913_2024_12178_MOESM1_ESM.docx]

**Table S1. Descriptive analysis of visits to physicians and nurses in Primary Care during the different study years in the profiles of patients with hypertension, type 2 DM and dyslipidaemia in the CARhES cohort.**

| **Hypertension** | | |  | | | | | |  | | | | |  | | | | | | | | |  | | | | | | | | | |  | | | | | | | | | | |  | | | | | | | | | |  | | | | | | | | |  |
| --- | --- | --- | --- | --- | --- | --- | --- | --- | --- | --- | --- | --- | --- | --- | --- | --- | --- | --- | --- | --- | --- | --- | --- | --- | --- | --- | --- | --- | --- | --- | --- | --- | --- | --- | --- | --- | --- | --- | --- | --- | --- | --- | --- | --- | --- | --- | --- | --- | --- | --- | --- | --- | --- | --- | --- | --- | --- | --- | --- | --- | --- | --- | --- |
|  | |  | **Profile 1** | | | | | | **Profile 2** | | | | | **Profile 3** | | | | | | | | | **Profile 4** | | | | | | | | | | **Profile 5** | | | | | | | | | | | **Profile 6** | | | | | | | | | | **Profile 7** | | | | | | | | |  |
|  | |  | **Mean** | | | **SD** | **Median** | | **Mean** | | **SD** | **Median** | | **Mean** | | **SD** | **Median** | | | | | | **Mean** | | **SD** | | | | **Median** | | | | **Mean** | | **SD** | | | | **Median** | | | | | **Mean** | **SD** | | | | **Median** | | | | | **Mean** | **SD** | | | **Median** | | | | |  |
| **Physician** | | 2017 | 6,9 | | | 7,7 | 5 | | 7,2 | | 7,8 | 5 | | 9,0 | | 8,6 | 7 | | | | | | 8,6 | | 6,8 | | | | 7 | | | | 10,0 | | 7,6 | | | | 8 | | | | | 11,3 | 8,7 | | | | 9 | | | | | 11,4 | 8,6 | | | 9 | | | | |  |
|  |  | 2018 | 6,7 | | | 7,7 | 5 | | 7,2 | | 7,8 | 5 | | 9,1 | | 8,7 | 7 | | | | | | 8,9 | | 7,1 | | | | 7 | | | | 10,1 | | 7,7 | | | | 8 | | | | | 11,6 | 8,9 | | | | 10 | | | | | 11,5 | 8,8 | | | 9 | | | | |  |
|  |  | 2019 | 6,8 | | | 7,9 | 5 | | 7,3 | | 8,0 | 5 | | 9,1 | | 8,7 | 7 | | | | | | 8,8 | | 7,1 | | | | 7 | | | | 10,1 | | 7,7 | | | | 8 | | | | | 11,4 | 8,9 | | | | 9 | | | | | 11,3 | 8,8 | | | 9 | | | | |  |
|  |  | 2020 | 7,6 | | | 9,0 | 5 | | 7,7 | | 8,8 | 5 | | 9,4 | | 9,5 | 7 | | | | | | 8,3 | | 7,5 | | | | 6 | | | | 9,8 | | 8,5 | | | | 8 | | | | | 11,0 | 9,4 | | | | 9 | | | | | 10,9 | 9,2 | | | 9 | | | | |  |
|  |  | 2021 | 7,6 | | | 8,7 | 5 | | 7,8 | | 8,2 | 6 | | 9,6 | | 9,2 | 7 | | | | | | 8,7 | | 7,3 | | | | 7 | | | | 10,4 | | 8,2 | | | | 8 | | | | | 11,1 | 9,1 | | | | 9 | | | | | 11,2 | 8,9 | | | 9 | | | | |  |
| **Nurse** | | 2017 | 2,5 | | | 5,2 | 1 | | 3,5 | | 6,8 | 1 | | 4,0 | | 6,7 | 2 | | | | | | 7,0 | | 10,1 | | | | 5 | | | | 7,0 | | 9,2 | | | | 5 | | | | | 10,3 | 13,1 | | | | 7 | | | | | 9,3 | 13,2 | | | 6 | | | | |  |
|  |  | 2018 | 2,3 | | | 5,3 | 1 | | 3,5 | | 8,9 | 1 | | 3,9 | | 6,4 | 2 | | | | | | 7,1 | | 10,1 | | | | 5 | | | | 7,0 | | 9,6 | | | | 5 | | | | | 10,5 | 13,6 | | | | 7 | | | | | 9,4 | 13,4 | | | 6 | | | | |  |
|  |  | 2019 | 2,3 | | | 4,9 | 1 | | 3,6 | | 7,2 | 1 | | 4,1 | | 6,6 | 2 | | | | | | 7,2 | | 10,7 | | | | 5 | | | | 7,2 | | 10,0 | | | | 5 | | | | | 10,3 | 13,4 | | | | 7 | | | | | 9,3 | 13,9 | | | 6 | | | | |  |
|  |  | 2020 | 2,5 | | | 5,3 | 1 | | 3,5 | | 6,6 | 2 | | 3,8 | | 5,8 | 2 | | | | | | 6,5 | | 9,6 | | | | 4 | | | | 6,4 | | 9,2 | | | | 4 | | | | | 9,5 | 13,0 | | | | 6 | | | | | 8,6 | 13,0 | | | 5 | | | | |  |
|  |  | 2021 | 2,7 | | | 5,1 | 1 | | 3,8 | | 7,0 | 2 | | 4,0 | | 6,1 | 2 | | | | | | 6,5 | | 9,9 | | | | 4 | | | | 6,5 | | 9,5 | | | | 4 | | | | | 9,7 | 13,4 | | | | 6 | | | | | 8,8 | 13,3 | | | 5 | | | | |  |
|  | |  |  | | |  |  | |  | |  |  | |  | |  |  | | | | | |  | |  | | | |  | | | |  | |  | | | |  | | | | |  |  | | | |  | | | | |  |  | | |  | | | | |  |
|  | |  |  | | | | | |  | | | | |  | | | | | | | | |  | | | | | | | | | |  | | | | | | | | | | |  | | | | | | | | | |  | | | | | | | | |  |
| **Type 2 DM** | |  | **Profile 1** | | | | | | **Profile 2** | | | | | **Profile 3** | | | | | | | | | **Profile 4** | | | | | | | | | | **Profile 5** | | | | | | | | | | | **Profile 6** | | | | | | | | | | **Profile 7** | | | | | | | | |  |
|  | |  | **Mean** | | **SD** | | **Median** | | **Mean** | | **SD** | **Median** | | **Mean** | | **SD** | | | | **Median** | | | **Mean** | | | **SD** | | | | **Median** | | | **Mean** | | | **SD** | | | | **Median** | | | | **Mean** | | | | **SD** | | | **Median** | | | **Mean** | | | **SD** | | | **Median** | | |  |
| **Physician** | | 2017 | 7,6 | | 7,4 | | 6 | | 8,2 | | 8,3 | 6 | | 10,3 | | 8,8 | | | | 8 | | | 9,4 | | | 7,1 | | | | 8 | | | 11,3 | | | 8,1 | | | | 9 | | | | 12,1 | | | | 9,2 | | | 10 | | | 12,3 | | | 9,1 | | | 10 | | |  |
|  |  | 2018 | 7,4 | | 7,5 | | 5 | | 8,3 | | 8,3 | 6 | | 10,4 | | 9,3 | | | | 8 | | | 9,7 | | | 7,3 | | | | 8 | | | 11,4 | | | 8,3 | | | | 10 | | | | 12,3 | | | | 9,4 | | | 10 | | | 12,4 | | | 9,0 | | | 10 | | |  |
|  |  | 2019 | 7,7 | | 7,8 | | 6 | | 8,4 | | 8,4 | 6 | | 10,5 | | 9,5 | | | | 8 | | | 9,7 | | | 7,5 | | | | 8 | | | 11,3 | | | 8,2 | | | | 10 | | | | 12,0 | | | | 9,2 | | | 10 | | | 12,1 | | | 9,0 | | | 10 | | |  |
|  |  | 2020 | 9,2 | | 9,9 | | 6 | | 8,7 | | 9,0 | 6 | | 11,0 | | 10,5 | | | | 8 | | | 9,3 | | | 8,0 | | | | 7 | | | 11,0 | | | 9,2 | | | | 9 | | | | 11,6 | | | | 9,9 | | | 9 | | | 11,8 | | | 9,4 | | | 9 | | |  |
|  |  | 2021 | 8,9 | | 9,2 | | 6 | | 8,9 | | 8,5 | 7 | | 11,0 | | 9,6 | | | | 9 | | | 9,7 | | | 7,8 | | | | 8 | | | 11,6 | | | 8,7 | | | | 10 | | | | 11,7 | | | | 9,5 | | | 10 | | | 12,2 | | | 9,4 | | | 10 | | |  |
| **Nurse** | | 2017 | 5,1 | | 6,5 | | 3 | | 5,5 | | 10,5 | 3 | | 6,3 | | 8,1 | | | | 4 | | | 8,8 | | | 11,5 | | | | 6 | | | 9,5 | | | 11,1 | | | | 7 | | | | 12,0 | | | | 14,7 | | | 8 | | | 11,6 | | | 14,6 | | | 8 | | |  |
|  |  | 2018 | 4,9 | | 6,1 | | 3 | | 5,6 | | 10,0 | 3 | | 6,4 | | 8,5 | | | | 4 | | | 9,0 | | | 11,1 | | | | 7 | | | 9,6 | | | 11,6 | | | | 7 | | | | 12,1 | | | | 14,6 | | | 8 | | | 11,6 | | | 15,0 | | | 8 | | |  |
|  |  | 2019 | 5,5 | | 6,9 | | 3 | | 5,8 | | 10,6 | 3 | | 6,5 | | 7,9 | | | | 5 | | | 9,2 | | | 11,7 | | | | 7 | | | 9,8 | | | 11,9 | | | | 7 | | | | 12,0 | | | | 14,3 | | | 8 | | | 11,5 | | | 15,0 | | | 8 | | |  |
|  |  | 2020 | 7,0 | | 7,9 | | 4 | | 5,8 | | 9,9 | 3 | | 6,5 | | 7,9 | | | | 4 | | | 8,6 | | | 10,9 | | | | 6 | | | 8,9 | | | 10,6 | | | | 6 | | | | 10,9 | | | | 13,4 | | | 7 | | | 10,7 | | | 14,1 | | | 7 | | |  |
|  |  | 2021 | 7,2 | | 8,1 | | 5 | | 6,2 | | 10,7 | 4 | | 6,7 | | 8,8 | | | | 4 | | | 8,5 | | | 11,2 | | | | 6 | | | 9,0 | | | 11,0 | | | | 6 | | | | 11,3 | | | | 14,2 | | | 8 | | | 11,0 | | | 14,6 | | | 7 | | |  |
|  | |  | | | | |  | | | | |  | | | | | | | | |  | | | | | | | | | |  | | | | | | | | | | |  | | | | | | | | | |  | | | | | | | | |  | | |
| **Dyslipidaemia** | | **Profile 1** | | | | | **Profile 2** | | | | | **Profile 3** | | | | | | | | | **Profile 4** | | | | | | | | | | **Profile 5** | | | | | | | | | | | **Profile 6** | | | | | | | | | | **Profile 7** | | | | | | | | | **Profile 8** | | |
|  |  | **Mean** | **SD** | **Median** | | | **Mean** | **SD** | | **Median** | | **Mean** | **SD** | | **Median** | | | | **Mean** | | | **SD** | | **Median** | | | | **Mean** | | | | **SD** | | **Median** | | | | **Mean** | | | **SD** | | **Median** | | | **Mean** | | | | **SD** | | | **Median** | | | **Mean** | | | **SD** | | | **Median** | |
| **Physician** | 2017 | 5,0 | 6,4 | 3 | | | 7,1 | 7,4 | | 5 | | 6,6 | 7,6 | | 5 | | | | 8,3 | | | 8,1 | | 6 | | | | 8,4 | | | | 6,6 | | 7 | | | | 9,7 | | | 7,4 | | 8 | | | 11,4 | | | | 8,5 | | | 9 | | | 11,4 | | | 8,4 | | | 9 | |
|  | 2018 | 4,9 | 6,5 | 3 | | | 7.0 | 7,5 | | 5 | | 6,7 | 7,6 | | 5 | | | | 8,5 | | | 8,4 | | 6 | | | | 8,7 | | | | 6,9 | | 7 | | | | 9,7 | | | 7,4 | | 8 | | | 11,8 | | | | 8,9 | | | 10 | | | 11,6 | | | 8,7 | | | 10 | |
|  | 2019 | 4,9 | 6,4 | 3 | | | 7,2 | 7,8 | | 5 | | 6,8 | 7,7 | | 5 | | | | 8,6 | | | 8,4 | | 6 | | | | 8,6 | | | | 6,9 | | 7 | | | | 9,7 | | | 7,5 | | 8 | | | 11,5 | | | | 8,8 | | | 9 | | | 11,3 | | | 8,6 | | | 9 | |
|  | 2020 | 5,7 | 7,4 | 3 | | | 8,1 | 9,2 | | 5 | | 7,2 | 8,5 | | 5 | | | | 9,0 | | | 9,4 | | 6 | | | | 8,2 | | | | 7,4 | | 6 | | | | 9,4 | | | 8,3 | | 7 | | | 11,0 | | | | 9,3 | | | 9 | | | 10,9 | | | 9,1 | | | 9 | |
|  | 2021 | 5,8 | 7,3 | 4 | | | 8,5 | 8,9 | | 6 | | 7,5 | 8,2 | | 5 | | | | 9,3 | | | 9,0 | | 7 | | | | 8,6 | | | | 7,2 | | 7 | | | | 10,0 | | | 8,1 | | 8 | | | 11,1 | | | | 8,9 | | | 9 | | | 11,2 | | | 8,7 | | | 9 | |
| **Nurse** | 2017 | 1,4 | 3,6 | 0 | | | 1,7 | 3,6 | | 1 | | 2,7 | 6,1 | | 1 | | | | 2,9 | | | 5,5 | | 1 | | | | 6,4 | | | | 9,4 | | 4 | | | | 6,0 | | | 8,5 | | 4 | | | 10,1 | | | | 12,9 | | | 7 | | | 8,9 | | | 12,2 | | | 6 | |
|  | 2018 | 1,3 | 3,6 | 0 | | | 1,7 | 3,7 | | 0 | | 2,8 | 6,2 | | 1 | | | | 2,9 | | | 5,5 | | 1 | | | | 6,5 | | | | 9,2 | | 4 | | | | 6,1 | | | 9,0 | | 4 | | | 10,2 | | | | 13,3 | | | 7 | | | 9,0 | | | 12,4 | | | 6 | |
|  | 2019 | 1,4 | 3,8 | 0 | | | 1,9 | 4,0 | | 1 | | 3,0 | 6,5 | | 1 | | | | 3,1 | | | 5,6 | | 1 | | | | 6,7 | | | | 9,7 | | 4 | | | | 6,3 | | | 9,3 | | 4 | | | 10,2 | | | | 13,0 | | | 7 | | | 9,0 | | | 13,0 | | | 5 | |
|  | 2020 | 1,8 | 4,2 | 0 | | | 2,3 | 4,2 | | 1 | | 3,0 | 6,1 | | 1 | | | | 3,1 | | | 5,1 | | 2 | | | | 6,2 | | | | 8,7 | | 4 | | | | 5,7 | | | 8,3 | | 3 | | | 9,3 | | | | 12,7 | | | 5 | | | 8,2 | | | 12,1 | | | 4 | |
|  | 2021 | 1,9 | 4,0 | 1 | | | 2,4 | 4,2 | | 1 | | 3,3 | 6,3 | | 1 | | | | 3,3 | | | 5,5 | | 2 | | | | 6,2 | | | | 9,4 | | 3 | | | | 5,7 | | | 8,6 | | 3 | | | 9,6 | | | | 12,9 | | | 6 | | | 8,4 | | | 12,6 | | | 5 | |
|  |  |  |  |  | | |  |  | |  | |  |  | |  | | | |  | | |  | |  | | | |  | | | |  | |  | | | |  | | |  | |  | | |  | | | |  | | |  | | |  | | |  | | |  | |
|  | |  | | | | |  | | | | |  | | | | | |  | | | | | | | | |  | | | | | | | | | |  | | | | | | | | | |  | | | | | | | |  | | | | | | | | |

SD: Standard Deviation; NA: Not Applicable because there are not enough observations for the estimation.

**Table S2. Number (%) of subjects with at least one hospitalisation and their mean (SD) number of hospitalisations. Results stratified by sex, CVD risk factor and tertiles of morbidity burden. CARhES cohort, 2017.**

|  |  |  | **Total** | **Hypertension** | **Type 2 DM** | **Dyslipidaemia** |
| --- | --- | --- | --- | --- | --- | --- |
| **N (%) subjects with ≥ 1 hospitalisation** | *T1* | *Men* | 5,236 (2.4) | 2,301 (1.9) | 2,061 (3.9) | 3,936 (2.4) |
|  |  | *Women* | 6,056 (2.7) | 2,567 (1.9) | 2,092 (4.8) | 4,114 (2.5) |
|  | *T2* | *Men* | 8,464 (3.8) | 4,947 (4.1) | 2,580 (4.9) | 6,296 (3.8) |
|  |  | *Women* | 8,351 (3.7) | 4,864 (3.7) | 1,971 (4.5) | 5,972 (3.6) |
|  | *T3* | *Men* | 20,719 (9.4) | 12,066 (10.1) | 6,138 (11.6) | 15,146 (9.1) |
|  |  | *Women* | 18,818 (8.3) | 11,799 (8.9) | 4,626 (10.6) | 13,345 (8.0) |
| **Mean (SD) number of hospitalisations** | *T1* | *Men* | 1.3 (0.9) | 1.2 (0.7) | 1.5 (1.0) | 1.3 (0.8) |
|  |  | *Women* | 1.3 (0.7) | 1.2 (0.6) | 1.4 (0.8) | 1.3 (0.7) |
|  | *T2* | *Men* | 1.4 (1.0) | 1.3 (1.0) | 1.5 (1.0) | 1.4 (1.0) |
|  |  | *Women* | 1.3 (0.7) | 1.3 (0.7) | 1.4 (0.9) | 1.3 (0.9) |
|  | *T3* | *Men* | 1.7 (1.3) | 1.7 (1.2) | 1.8 (1.4) | 1.7 (1.3) |
|  |  | *Women* | 1.6 (1.1) | 1.6 (1.1) | 1.7 (1.3) | 1.6 (1.1) |

DM: Diabetes Mellitus
